# Susceptibility Factors in Chronic Lung Inflammatory Responses to Engineered Nanomaterials

**DOI:** 10.3390/ijms21197310

**Published:** 2020-10-03

**Authors:** Dorothy J. You, James C. Bonner

**Affiliations:** Toxicology Program, Department of Biological Sciences, North Carolina State University, Raleigh, NC 27695, USA; djyou@ncsu.edu

**Keywords:** engineered nanomaterials, susceptibility, chronic inflammation, lung inflammation.

## Abstract

Engineered nanomaterials (ENMs) are products of the emerging nanotechnology industry and many different types of ENMs have been shown to cause chronic inflammation in the lungs of rodents after inhalation exposure, suggesting a risk to human health. Due to the increasing demand and use of ENMs in a variety of products, a careful evaluation of the risks to human health is urgently needed. An assessment of the immunotoxicity of ENMs should consider susceptibility factors including sex, pre-existing diseases, deficiency of specific genes encoding proteins involved in the innate or adaptive immune response, and co-exposures to other chemicals. This review will address evidence from experimental animal models that highlights some important issues of susceptibility to chronic lung inflammation and systemic immune dysfunction after pulmonary exposure to ENMs.

## 1. Introduction

Inflammation is the consequence of an innate immune response of the host to stimuli such as pathogens (e.g., bacteria, fungi, viruses), allergens, or toxic chemicals and pollutants [1,2,3]. The steps in the inflammatory response can be defined as (1) induction, (2) peak, and (3) resolution [1,2,4,5,6,7]. The induction phase involves innate immune cells (e.g., macrophages) detecting the pathogen or other foreign agent and orchestrating the recruitment of other innate immune cells (e.g., neutrophils, eosinophils) to the site of infection and/or injury [1,2,5,6,7]. The peak of inflammation involves recruited innate immune cells (e.g., macrophages, monocytes, neutrophils) engulfing pathogens or inhaled agents, triggering key defensive mechanisms, notably the generation of reactive oxygen species (i.e., respiratory burst) from macrophages and the release of neutrophil extracellular traps (NETs) from recruited neutrophils [1,2,3,4,5,6,7]. Recruited innate immune cells also secrete a plethora of cytokines (e.g., IL-6, IL-1β, TNF-α) and chemokines (e.g., CXC and CC motif chemokines) that have numerous functions (e.g., cell proliferation, cell migration, cell death, alteration of epithelial or endothelial barrier permeability) and also serve as extracellular signals that bridge the innate and adaptive immune systems. The resolution of inflammation involves clearance of foreign agents, removal of recruited host immune cells, and repair of damaged tissue [8,9]. During an acute inflammatory response, the body’s innate immune system defends against the foreign agent in order to remove the invading threat and to heal the damaged tissue through the recruitment of various immune cells such as neutrophils, monocytes, macrophages, lymphocytes, and plasma cells [1,2,3,4,5,6,7,8,9]. The induction of acute inflammation is a rapid process that occurs within hours and resolves within days, where the immune process is more localized to the specific injury site [9,10]. This acute inflammatory response is critical to restore the body back to a state of homeostasis after injury [5,6,7,8,9,10]. However, when acute inflammation fails to resolve or multiple exposures dysregulate the immune system, this may lead to unresolved chronic inflammation [4,5,6,7,8,9,10].

Unresolved chronic inflammation involves persistent activation of the innate immune response, over a period of weeks, months, or years, as well as involvement of the adaptive immune response [11,12,13]. While the innate immune system initiates the acute inflammatory response toward a foreign agent, the adaptive immune system is a more complicated system involving the recognition of a specific antigen, the polarization and clonal expansion of specific lymphocyte populations, and the production of antibodies toward the specific antigen [14,15]. The adaptive immune system is not an immediate response but provides long-lasting ‘memory’ to the foreign agent [14,15]. Both the innate and adaptive immune systems are closely connected to each other and both components are involved in unresolved chronic inflammation that culminates in a variety of disease states, including allergies, asthma, fibrosis, and cancer [16,17,18,19]. Unresolved chronic lung inflammation would sustain higher numbers of inflammatory cells and mediators to reside around the inflammatory site, worsening tissue injury and prolonging the cascade of pro-inflammatory signaling. One of the most well-known lung diseases that results from chronic inflammation in the lung is asthma [14,19]. Asthma is a chronic inflammatory disorder that is characterized by airway hyperresponsiveness (AHR) and airway remodeling, both of which reduce lung function [14,20]. Asthma affects approximately 300 million people worldwide and 26 million people in the United States [21,22]. Importantly, asthma is exacerbated by a variety of agents, including ultrafine air pollution particles (i.e., ambient nanoparticles). Thus, there is a critical need to elucidate the mechanisms of unresolved chronic inflammation in chronic lung diseases such as asthma that are exacerbated by inhaled nanoparticles. 

Engineered nanomaterials (ENMs) are increasingly incorporated into a variety of products, making human exposure inevitable [23,24,25,26,27,28,29,30,31]. ENMs are defined as a class of engineered substances with at least one dimension in the range of 1–100 nm [32,33]. The high surface area per mass ratio and other physicochemical characteristics impart novel properties for use of ENMs in diverse fields including catalysis, electronics, textile fabrication, personal cosmetics, paints, and other various industrial applications [25,29,33,34,35,36,37]. In recent years, ENMs have gained increasing attention in the field of nanomedicine for the diagnosis and treatment of a variety of diseases, including cancer [38,39,40,41]. However, due to the small size and high reactivity of many ENMs, there is the risk of possible adverse human health effects upon exposure [29]. There are many different types of ENMs used for industrial applications including metal nanoparticles and carbon nanoparticles (e.g., carbon nanotubes (CNTs), carbon nanofibers (CNFs), and graphene), as well as those used for nanomedicine applications such as polymeric nanoparticles, solid lipid nanoparticles, and liposomes [25,33,38,42]. CNTs make up a big portion of the nanotechnology market. Market analysis predicts that CNTs will represent a multi-billion dollar industry within the next five years [43]. CNTs include single-walled carbon nanotubes (SWCNTs) resembling a rolled graphene sheet with a diameter between 1 and 4 nm and multi-walled carbon nanotubes (MWCNTs) containing multiple rolled graphene sheets with diameters between 10 and 100 nm [44,45,46,47]. Different CNTs are utilized in electronics, medical applications, battery manufacturing, and polymers [48,49,50]. 

There is growing evidence from in vitro cell and in vivo rodent models that different ENMs can interact with the immune system [51,52,53,54,55,56,57,58,59]. For example, it has been found that macrophage activation can be dysregulated by silica nanoparticles [60]. In another study, it was suggested that silver nanoparticles (AgNPs) influenced gut homeostasis by compromising barrier integrity in intestinal epithelial cells co-cultured with macrophages [61]. That work also showed that an inflamed condition, simulated by the addition of LPS and IFN-γ in vitro, caused greater AgNP-induced epithelial necrosis and apoptosis. Other reports have demonstrated that different types of ENMs such as CNTs or fullerenes disrupt the function of immune cells including B cells, NK cells, mast cells, and macrophages [62,63]. The greatest exposure route for ENMs is through inhalation, leading to concern that ENMs will cause or exacerbate respiratory diseases. Many previous studies have shown that ENMs cause chronic lung inflammation, pulmonary fibrosis, lung cancer, or exacerbation of allergic lung disease in experimental animals [56,64,65,66,67,68,69,70,71,72,73,74,75]. Other studies using cultured human or rodent lung cells demonstrate that ENMs increase the production of pro-inflammatory or pro-fibrotic mediators [76,77]. There is some limited evidence that occupational ENM exposure (i.e., MWCNTs) is associated with increased pro-fibrotic mediators in serum and induced sputum of workers [74]. Therefore, repeated pulmonary exposures to ENMs may possess a new risk for chronic unresolved lung inflammation in humans. 

**Figure 1:**
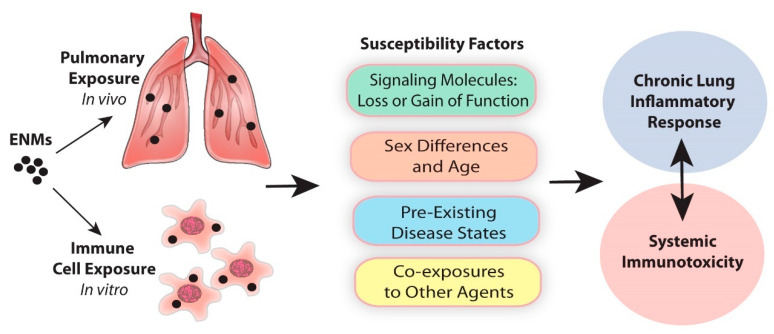
Susceptibility factors in the chronic lung inflammatory response to engineered nanomaterials (ENMs).

There is a large gap of knowledge in terms of which susceptibility factors are most important in determining the severity of ENM-induced chronic respiratory diseases in humans, such as asthma, fibrosis, and cancer. Therefore, in this review, we will summarize some of the susceptibility factors that appear to be important in determining the severity of lung inflammatory responses and systemic immunotoxicity in experimental animals exposed to ENMs. These susceptibility factors include deficiency or dysregulation of specific genes, sex, pre-existing diseases, and co-exposures to other chemicals, allergens, or microbial agents that may increase the severity of chronic unresolved inflammatory responses of the lung to ENMs (Figure 1). In addition, we will identify some gaps in our understanding of susceptibility to ENM-induced lung inflammation that should help to better predict the risks to human health. 

## 2. Deficiency in Cell Signaling Molecules as Determinants of Susceptibility to ENM-Induced Chronic Lung Inflammation

### 2.1. Transcription Factors

#### 2.1.1. STAT1

The signal transducers and activators of transcription (STATs) are a family of seven transcription factors that remain inactivated in the cytoplasm until activation by extracellular signaling proteins like cytokines or growth factors binding to their cognate receptors on the cell surface [78]. Among them, STAT1 is known as a regulator of apoptosis, growth arrest, and development and maintenance of T helper 1 (T_H_1) cells [78,79]. Therefore, the actions of STAT1 are critical to oppose the development of T helper 2 (T_H_2) cells. It has been previously shown that *Stat*1 Knockout (KO) mice are susceptible to mortality caused by viral and bacterial infection, primarily due to the fact that the STAT1 pathway is a major signaling pathway through which interferons (IFNs) mediate antiviral activity [80]. *Stat*1 KO mice are also susceptible to bleomycin-induced lung fibrosis with heightened proliferative responses to growth factors such as platelet-derived growth factor (PDGF) and epidermal growth factor (EGF) [81]. As summarized in Table 1, *Stat1* KO mice exhibit increased fibrosis induced by rigid MWCNTs with higher levels of TGF-β1 in the bronchoalveolar lavage fluid (BALF) and increased Smad2/3 phosphorylation in lung tissue [82]. Furthermore, STAT1 plays a critical role to protect against allergen-induced airway remodeling and exacerbation by tangled MWCNTs [83]. From a clinical perspective, patients with idiopathic pulmonary fibrosis have lower levels of STAT1 protein than normal individuals [84]. Therefore, STAT1 deficiency likely plays an important role in lung inflammation that results in chronic lung diseases and can be a risk factor to increase susceptibility toward ENMs.

#### 2.1.2. T-bet

T-box transcription factor TBX21 (T-bet) is a transcription factor that plays a critical role in the development and differentiation of T_H_1 cells in the lung [85]. T-bet regulates IFN-γ production and inhibits T_H_2 cell development [85]. It has been reported that mice with targeted deletion of T-bet have decreased production of IFN-γ and increased production of T_H_2 cytokines, such as IL-4 and IL-13, leading to the spontaneous development of allergic airway remodeling (eosinophilic infiltration, airway mucous cell metaplasia, and subepithelial fibrosis) similar to pathological changes observed in the lungs of asthma patients [86]. As summarized in Table 1, homozygous *T-bet* KO mice exhibit enhanced mucous cell metaplasia three weeks after exposure to nickel nanoparticles (NiNPs) compared to wild type mice [87]. The enhanced mucous cell metaplasia in *T-bet* KO mice induced by NiNPs was accompanied by an increase in MUC5AC and MUC5B mRNAs in the lung [87]. Furthermore, numbers of inflammatory cells, including eosinophils and lymphocytes, persisted in the BALF of *T-bet* KO mice for at least three weeks after the final NiNP exposure [87]. Moreover, *T-bet* KO mice developed more interstitial lung fibrosis after NiNP exposure compared to wild type mice [87]. MWCNT exposure via oropharyngeal aspiration also increased mucous cell metaplasia in *T-bet* KO compared to the wildtype mice, but to a lesser extent compared to NiNPs [87]. Therefore, T-bet appears to play an important role in the polarization of T cells towards a T_H_1 phenotype and suppression of allergic T_H_2 lung inflammation. Since *T-bet* KO mice are susceptible to chronic allergic lung inflammation caused by NiNPs and MWCNTs, this suggests that individuals with T-bet deficiency would be more susceptible to certain types of ENMs. 

#### 2.1.3. Nrf2

Nuclear factor erythroid 2-related factor (Nrf2) is a transcription factor that controls the expression of antioxidant proteins [88]. Nrf2 is activated to increase the production of antioxidants such as drug-metabolizing enzymes including glutathione S-transferase and NAD(P)H: quinone oxidoreductase 1 upon exposure to oxidative stress in cells [88]. Activation of Nrf2 has been implicated as a protective factor that counteracts the pathogenesis of lung disease in mice and humans upon inhaled oxidants including ozone, cigarette smoke, and air pollution particles [89]. It has been reported that a lack of Nrf2 results in a severe outcome inpatients with respiratory infections, chronic obstructive pulmonary disease (COPD), asthma, idiopathic pulmonary fibrosis, and lung cancer [89]. As summarized in Table 1, exposure of *Nrf2* KO mice to MWCNTs by oropharyngeal aspiration showed a higher level of inflammation and fibrosis with increased inflammatory cell infiltrates in the lungs 7 days after the initial exposure compared to the wild type mice [90]. Moreover, increased ROS generation, oxidative damage, and lung inflammation in *Nrf2* KO mice exposed to MWCNTs suggests that Nrf2 is critical towards suppressing the lung inflammatory response [90]. Another study, summarized in Table 1, showed that *Nrf2* KO mice exposed to silica nanoparticles via intranasal instillation displayed increased generation of reactive oxygen species (ROS) and decreased total antioxidant capacity compared to wild type mice [91]. This study also suggested that Nrf2 protects against the oxidative stress induced by silica nanoparticles [91]. Therefore, the role of Nrf2 in controlling antioxidant protein expression may be critical to resolve inflammatory responses through suppression of ROS generated by ENMs. The source of these ROS could be directly generated from ENMs via surface chemistry, or indirectly by inflammatory cells (e.g., macrophages) undergoing a respiratory burst in response to ENM exposure. 

#### 2.1.4. P53

P53 is a transcription factor and tumor suppressor that is pivotal in the progression of lung cancer and also appears to play a role in pulmonary fibrosis [92]. For example, idiopathic pulmonary fibrosis patients have a higher incidence of mutated p53 genes and overexpression of mutated p53 [93,94]. Human lung epithelial (BEAS-2B) cells exposed to SWCNTs for 12–24 weeks showed increased resistance to apoptosis and a decrease in p53 activation in vitro [95]. Furthermore, mesothelioma formation in the pleural lining of the abdominal cavity in p53 heterozygous mice (*p53*^+/-^) has been reported following intraperitoneal injection of MWCNTs [96]. However, that study lacked a comparison to wild type *p53*^+/+^ mice. As summarized in Table 1, an increased incidence of larger granulomas, lymphoid aggregates, and epithelial cell hyperplasia was observed in the lungs of *p53*^+/-^ mice, compared to wild type mice, eleven months after oropharyngeal exposure to rigid (Mitsui-7) MWCNTs [97]. Therefore, p53 could play a role in suppressing chronic lung inflammation and granuloma formation upon pulmonary exposures to different ENMs, including SWCNTs and MWCNTs. Deficiency of p53 in experimental animals and humans is a susceptibility factor in pulmonary diseases, including cancer and pulmonary fibrosis. 

#### 2.1.5. BMAL1

Brain and muscle ARNT-like protein (BMAL1) is a transcription factor that controls circadian rhythm and regulates ROS generation [98]. *Bmal1* KO mice showed higher platelet aggregation and adhesion, indicating a higher risk for cardiovascular disease compared to wild type mice [99]. Moreover, most of the *Bmal1* KO mice die at an age between 26 and 52 weeks, mainly due to excessive ROS production and a chronic oxidative stress state in various tissues, resembling an early aging phenotype [100]. As summarized in Table 1, both wild type and *Bmal1* KO mice have been evaluated for pro-inflammatory responses after exposure to zinc oxide nanoparticles (ZnONPs) or MWCNTs delivered to the lungs via oropharyngeal aspiration over 5 weeks [98]. MWCNT exposure caused an increase in inflammatory responses, cell counts in BALF, oxidative stress, and procoagulant effects in the serum of *Bmal1* KO mice compared to wild type mice [98]. On the other hand, ZnONP exposure showed a decrease in inflammatory and oxidative responses but increased the procoagulant effect in *Bmal1* KO mice compared to the wild type mice [98]. Although MWCNTs and ZnONPs produced opposite inflammatory responses in *Bmal1* KO mice, the dysregulation of coagulation induced by both of these ENMs in *Bmal1* KO mice indicates that BMAL1 plays an important role in preventing adverse cardiovascular effects of ENMs [98]. However, different mechanisms appear to be involved in the chronic inflammatory responses to MWCNTs and ZnONPs.

### 2.2. Enzymes/Proteins

#### 2.2.1. NADPH Oxidase

NADPH oxidases are enzymes that are responsible for producing superoxide radicals (O_2_^•-^) [101]. Neutrophils kill invading pathogens by generating reactive oxygen species (ROS) such as O_2_^•-^ [101]. ROS have recently been shown to be a critical factor in controlling the resolution of inflammation by regulating neutrophils and macrophages by sending “eat-me” signals [101]. NADPH oxidase-deficient mice or *gp91*^phox−/−^ that specifically lacks the gp91^phox^ subunit of the enzyme have been used to evaluate lung inflammation after exposure to SWCNTs [102]. This study, summarized in Table 2, showed that *gp91*^phox−/−^ KO mice had augmented lung inflammation with higher numbers of neutrophils, apoptotic cells, pro-inflammatory cytokines (TNF-α, CCL2, and IL-6), and reduced TGF-β1 at 1, 7, and 28 days after pharyngeal exposure to SWCNTs [102]. The results also suggest that the impaired resolution of the inflammatory response may develop into a chronic unresolved inflammatory state, due to prolonged increase in neutrophils and pro-inflammatory cytokines, in NADPH oxidase deficient mice [102]. Thus, NADPH oxidase could play a protective role in the resolution of ENM-induced lung inflammation by releasing ROS to regulate neutrophils and macrophages. 

#### 2.2.2. COX-2

Cyclooxygenase-2 (COX-2), also known as prostaglandin-endoperoxide synthase-2 (PTGS2), is an enzyme that has been implicated in asthma and fibrosis [103]. Along with T_H_2 cytokines, including IL-13 and IL-5, COX-2 has been implicated in asthma pathogenesis [104,105]. However, unlike IL-13 and IL-5, which are increased in individuals with asthma, COX-2 deficiency has been linked to increased severity of asthma progression [103,104,105,106,107]. Particularly, individuals with asthma have a reduced level of mRNA encoding COX-2 in airway epithelial cells [106]. Furthermore, a study using airway epithelial cells collected from endobronchial brushings from healthy individuals, when treated with IL-13 to mimic the asthmatic microenvironment, had reduced COX-2 mRNA expression levels [107]. Lower levels of COX-2 in airway epithelial cells from the healthy individuals treated with IL-13 resulted in lower prostaglandin E_2_ (PGE_2_) [107]. Therefore, these data indicate that COX-2 plays a protective role in the asthmatic lung and loss of COX-2 and PGE_2_ results in susceptibility to allergic airway inflammation. As summarized in Table 2, *Ptgs2* KO mice sensitized to ovalbumin allergen through repeated intranasal aspiration followed by a single oropharyngeal aspiration of MWCNTs were found to be more susceptible to eosinophilic lung inflammation, airway mucous cell metaplasia, and airway fibrosis compared to wild type mice [103]. Compared to wild type mice, the *Ptgs2* KO mice also had significantly higher Th2 cytokines including IL-13, Th1 cytokines such as CXCL10, and the Th17 cytokine IL-17A [103]. Overall, this study showed that *Ptgs2* KO mice were susceptible to the exacerbation of allergen-induced airway disease by MWCNTs [103]. Deficiency in COX-2 could therefore be a possible mechanism involved in chronic lung inflammation induced by certain types of ENMs such as MWCNTs. Thus, COX-2 appears to be an enzyme that is needed for the resolution of chronic airway inflammation and loss of COX-2 could lead to susceptibility to ENM-induced lung inflammation.

#### 2.2.3. TIMP1

Tissue inhibitor of metalloproteinase 1 (TIMP1) is a glycoprotein that acts as an extracellular signaling molecule to control cell growth, apoptosis, differentiation, angiogenesis, and oncogenesis [108]. TIMP1 also plays a role to control extracellular matrix by mediating the activity of matrix metalloproteinases (MMPs) [108]. Furthermore, it has been reported that high expression of TIMP1 is associated with liver and pulmonary fibrosis in animal models [109,110,111]. It has been demonstrated that MWCNT-induced lung fibrosis in mice corresponded with highly upregulated TIMP1 at both the mRNA and protein level in lung tissue [112]. Moreover, *Timp1* KO mice exposed to MWCNTs as summarized in Table 2 had reduced lung fibrosis, suppressed myofibroblast differentiation, and lower activation of extracellular signal-regulated kinase (ERK) signaling, indicating that TIMP1 plays a pro-fibrotic role in the progression of MWCNT-induced lung fibrosis through activation of the intracellular ERK pathway [112]. Thus, TIMP1 may aggravate MWCNT-induced lung inflammation and may contribute to the susceptibility to chronic lung disease. 

#### 2.2.4. MPO

Myeloperoxidase (MPO) is an abundant enzyme in inflammatory cells especially produced by neutrophils and plays a vital defense role in the innate immune system [113]. Intratracheal instillation of MWCNTs and SWCNTs in rats has been shown to consistently increase the concentration of MPO in BALF [114]. Increased MPO was also associated with an increased number of total cells and neutrophils in the BALF, suggesting that it could be a potential biomarker for pulmonary toxicity induced by ENMs [114]. However, MPO has been reported to effectively mediate the oxidative biodegradation of CNTs (SWCNTs or MWCNTs), leading to resolution of inflammation [113]. To support this, *MPO* KO mice exposed to SWCNTs (summarized in Table 2) showed less efficient clearance of the nanomaterials [115]. This same study showed that *MPO* KO mice had a slightly weaker acute neutrophilic inflammatory response at day 1 after exposure to SWCNTs but significantly greater lung fibrosis at day 28. Thus, these findings suggest that increased MPO from pulmonary injury induced by ENMs could be playing a vital role in both initiating and resolving inflammation. As such, MPO has been suggested to be a double-edged sword where it can both be responsible for the initiation of inflammation and the resolution of inflammation caused by pulmonary exposure to ENMs. 

#### 2.2.5. ApoE

Apolipoprotein E (ApoE) is a protein that is responsible for fat metabolism in the body [116]. It has been reported that *ApoE* KO mice have increased vascular dysfunction to particulates like diesel exhaust and fullerene C60 (C60) nanoparticles [117,118]. As summarized in Table 2, intratracheal instillation of different ENMs, including carbon black nanoparticles (CBNPs), gold nanoparticles (AuNPs), C60 nanoparticles, and SWCNTs, increased DNA damage in BALF inflammatory cells, increased neutrophils in BALF, and higher BALF protein levels in *ApoE* KO mice compared to wild type mice [119]. *ApoE* KO mice with repeated exposure to MWCNTs via intratracheal instillation were also more susceptible to oxidative DNA damage in lung tissue compared to the wildtype mice [120]. Suzuki et al. also found that pharyngeal aspiration to single or double-walled CNTs caused endothelial progenitor cell (EPC) dysfunction and reduced migration function of EPCs in *ApoE* KO mice [121]. Dysregulation of EPCs in *ApoE* KO mice could contribute to the development of atherosclerosis [121]. Another study using *ApoE* KO mice treated with MWCNTs via intratracheal instillation showed a positive association between pulmonary inflammation and oxidative stress, along with the expression of genes involved in vascular activation [122]. Increased vascular activation from MWCNTs also could play a role in exacerbating the progression of atherosclerosis [122]. Overall, these studies suggest that a lack of ApoE is a susceptibility factor in ENM-induced lung inflammation and cardiovascular disease. 

### 2.3. Receptors

#### 2.3.1. AhR

Aryl hydrocarbon receptor (AhR) is a transcription factor that was originally shown to bind and become activated by environmental toxicants such as dioxins and polycyclic aromatic hydrocarbons [123]. Once activated, AhR transactivates phase I and phase II metabolizing enzymes such as cytochrome P450 1A1 [123]. Recent studies have shown that AhR is not only involved in metabolizing toxicants but may also be involved in the pulmonary immune and pro-inflammatory responses [124]. For example, a study using *AhR* KO mice exposed to ZnONPs via oropharyngeal aspiration (Table 3) showed that these mice had reduced cell numbers, total protein, LDH activity, and pro-inflammatory cytokine production in BALF compared to the wild type mice [125]. No increase in CYP1A1, a downstream target of AhR, was detected in *AhR* KO mice [125]. In addition, *AhR* KO mice did not display an increase in kynurenine (KYN), which is an endogenous AhR agonist [125]. Therefore, this work suggests that AhR is involved in mediating ZnONP-induced pulmonary inflammation. Furthermore, impaired/imbalanced AhR expression could be a potential factor in determining susceptibility to ENM-induced chronic lung inflammation.

#### 2.3.2. CCR5

CCR5 is a chemokine receptor that is known to mediate the T_H_1 response and binds several chemokines including RANTES/CCL5, MIP-1α, and MIP-1β [126,127]. A study by Park and colleagues, summarized in Table 3, compared the inflammatory response from *Ccr5* KO and wild type mice after exposure to SWCNTs via intratracheal instillation [128]. A single intratracheal instillation of SWCNTs significantly reduced the number of neutrophils in *Ccr5* KO mice, yet KO mice had more frequent histopathological lesions compared to the wildtype mice [128]. The lung inflammatory response of *Ccr5* KO mice was dominated by B cells and CD8+ T cells, while the wild type mice were mostly dominated by T cells and CD4+ T cells in the lungs [128]. Moreover, SWCNTs also increased IL-6, IL-13, and IL-17 in BALF of *Ccr5* KO mice compared to the wildtype mice [128]. These data suggested that the delay in the resolution of inflammation could be due to impaired cell migration to the inflammation site, which was supported by reduced numbers of neutrophils [128]. The authors of this work suggested that a delay in the resolution of inflammation resulted from shifting from a Th1 type response to a Th2 type response in the *Ccr5* KO model [128]. Overall, these findings suggest that *Ccr5* KO mice appear to be more susceptible to SWCNT-induced chronic lung inflammation due to the delay in the resolution of inflammation [128]. Therefore, CCR5 may play an important role in the resolution of inflammation induced by ENMs. 

### 2.4. Cytokines/Chemokines

#### 2.4.1. IL-1/Inflammasome

IL-1 is a family of cytokines that play a critical role in innate immunity [129]. There are 11 members in the family, and IL-1β, IL-1α, and IL-33 are the main IL-1 family members [129,130]. IL-1 family members provide non-specific immune responses toward foreign pathogens [129]. However, IL-1 also can mediate an adaptive immune response [129]. Therefore, IL-1 family members are pivotal cytokines that are involved in both acute and chronic inflammation [129]. Among the IL-1 family, the role of IL-1β has been studied the most extensively in ENM-induced lung inflammation. IL-1β secretion from inflammatory cells is regulated by inflammasomes, intracellular scaffolds that cleave pro-IL-1β to mature IL-1β when they detect extracellular signals [129]. The inflammasome is a critical complex to orchestrate IL-1 function [129]. For example, IL-1β is first produced as a precursor form, termed pro-IL-1β, that is lacking in biological activity [129]. In order to increase IL-1β production and secretion, the multiprotein complex inflammasome is assembled and serves as a docking scaffold for caspase-1 to cleave the inactive pro-IL-1β to active IL-1β. 

It has been shown that MWCNTs and other nanoparticles can activate the inflammasome assembly through lysosomal disruption [129,131]. For example, mice exposed with MWCNTs through oropharyngeal aspiration had increased IL-1β secretion in BALF [132]. Different studies have shown that IL-1α and IL-1β play roles in ENM-induced lung inflammation [131,132]. However, the role of IL-1β remains controversial, since some work has shown that reduced inflammasome activation results in greater pulmonary fibrosis induced by ENMs. For example, mice sensitized to house dust mites and challenged with MWCNTs showed reduced IL-1β in BALF but had more severe airway fibrosis after 21 days with increased pro-fibrogenic cytokines, including PDGF-A and PDGF-B mRNAs, compared to HDM or MWCNT treatment alone [132]. Another study, summarized in Table 3, showed that a 24 h acute exposure to MWCNTs, containing either low or high nickel content, through oropharyngeal aspiration in *IL-1R* KO mice had reduced acute inflammation and airway resistance but increased IL-6 protein production compared to the wild type mice [133]. However, this study also showed that the exposure to MWCNTs induced a significantly higher number of pulmonary granulomas and significant inflammation in *IL-1R* KO after 28 days compared to wild type mice [133]. The results of this study also showed that total inflammatory cells were reduced at both 24 h and 28 days post exposure in *IL-1R* KO mice [133]. It was suggested that the resolution of inflammation was inefficient in *IL-1R* KO mice, resulting in increased granuloma and inflammation after 28 days [133]. Similarly, another study also showed that acute inflammation induced by intratracheal instillation of MWCNTs was suppressed 24 h after the exposure in *IL-1R* KO mice, but fibrotic lesions still developed in KO mice after 28 days [134]. Other work using *IL-1R* KO mice exposed to rod-like MWCNTs by oropharyngeal aspiration (see Table 3) showed significantly reduced neutrophils in the BALF and lower levels of mRNA encoding CXCL5 (a neutrophil chemokine) at 4 h compared to the wildtype mice [135]. After the 28-day exposure to rod-like MWCNTs, neutrophils were still reduced in the BALF and TNF-α mRNA expression levels were suppressed compared to the wild type mice [135]. However, no changes were observed in T_H_2 related signals including mRNAs encoding IL-13 and TGF-β1, compared to the wild type mice [135]. Overall, the available evidence suggests that impaired IL-1 signaling caused by MWCNTs dysregulates the immune system, resulting in reduced acute inflammation or inefficient resolution of inflammation and resulting in pulmonary fibrosis and granuloma formation. 

#### 2.4.2. OPN

Osteopontin (OPN; secreted phosphoprotein 1 or SPP1) is a cytokine that is involved in various physiological and pathological processes, including inflammation, fibrosis, and bone remodeling [136]. Elevated OPN is expressed in tissues undergoing an intense wound healing process or during fibrogenesis [136]. It has been shown that both acute and chronic exposures to MWCNTs can induce higher OPN production levels [136]. A study using *Opn* KO mice showed that inhalation exposure to MWCNTs via pharyngeal aspiration (see Table 3) induced inflammation that was dependent on OPN [136]. The study also showed that *Opn* KO mice had reduced fibrotic formation and myofibroblast accumulation in the lungs compared to the wild type mice [136]. They suggested that OPN production and secretion in turn could activate TGF-β1 to promote fibrosis in the lungs [136]. Rats exposed to different doses of TiO_2_ particles via inhalation showed a significant increase in lung OPN mRNA and OPN protein in BALF in a dose–response manner [137]. Therefore, OPN appears to play a critical role in tissue remodeling. Individuals with excessive production of OPN might be more susceptible to unresolved chronic lung inflammation due to exposure to ENMs, resulting in excessive tissue remodeling and fibrosis in the lungs. 

#### 2.4.3. IL-6

Interleukin 6 (IL-6) is major pro-inflammatory cytokine and vital regulator for both the innate and adaptive immune system [138]. Studies have shown that IL-6 is increased systemically upon inhalation exposure to ENMs in mice and humans [139,140,141]. Specifically, pulmonary instillation of MWCNTs induced levels of systemic IL-6 in the heart, and MWCNTs coated with Zn also induced IL-6 mRNA levels in the heart and liver [141,142]. IL-6 can mediate both pro-inflammatory and anti-inflammatory functions [138]. An acute increase in IL-6 facilitates neutrophil recruitment to sites of tissue injury in order to induce acute inflammation [143]. However, IL-6 also activates STAT3 signaling which facilitates the reduction in neutrophils in acute inflammation [144]. Sustained IL-6 production also induces prolonged STAT3 activation and neutrophil recruitment to promote unresolved chronic inflammation [145]. Prolonged STAT3 activation has been implicated in many chronic inflammatory diseases including asthma, fibrosis, cancer, and hepatitis [145]. Therefore, adequate regulation of IL-6 is required for the initiation and resolution of inflammation. For example, a study showed that IL-6 can prevent the initiation but at the same time, promote the progression of lung cancer [146]. We recently reported that IL-6 is highly induced in the lungs of mice following acute exposure to NiNPs by oropharyngeal aspiration [147]. *Il6* KO mice have not yet been evaluated after inhalation exposure to ENMs. However, it is likely that IL-6 plays a major role in determining susceptibility to chronic inflammation. 

## 3. Sex

Sex can be a key determinant of susceptibility to ENM-induced chronic lung inflammation [148,149,150]. In this review, we will discuss this topic in the context of biological sex differences, including chromosome, sex organs, and endogenous hormonal profiles between males and females as defined by the Office of Research on Women’s Health at the National Institute of Health [151]. Sex is recognized as a critical factor in determining the susceptibility of individuals to respiratory disease [148,152,153,154,155,156,157,158,159,160,161,162,163]. Furthermore, according to epidemiology studies, there are significant differences in susceptibility between acute and chronic respiratory disorders [148,152,156,157,158,159,164]. Epidemiology studies show that men are more susceptible to acute lung inflammation from viral or bacterial infections and they have worse a prognosis compared to females [158,164,165]. As seen with the novel coronavirus SARS-CoV-2, epidemiology studies have found that a greater number of men are infected compared to women [166]. This trend was also seen in the MERS-CoV and SARS-CoV-1 viruses [166]. In contrast to men, women are more susceptible to developing chronic lung inflammation such as asthma [164,167]. Thus, clear susceptibility differences exist between men and women with regards to respiratory diseases, suggesting differences in pulmonary immune responses. 

To date, there is a lack of data depicting sex as a key determinant of susceptibility to ENM-induced chronic lung inflammation. However, there is some evidence showing susceptibility of either male or female rodents to ENM-induced lung inflammation. For example, Ray et al. found that a single dose of MWCNTs delivered via oropharyngeal aspiration caused a greater acute and chronic inflammatory response in female mice compared to male mice [148]. Kasai et al. also found that female rats were more susceptible to lung inflammation induced by rigid-MWCNTs via whole-body inhalation compared to male rats [168]. On the other hand, Ray and colleagues also showed that male mice were more susceptible to lung inflammation induced by repeated chronic exposure to crystalline silica (cSiO_2_) compared to female mice [148]. Others have shown that cSiO_2,_ delivered to the lungs of mice by intratracheal instillation, caused less severe lung inflammation in female mice than male mice [169,170]. Furthermore, it was demonstrated in the same study that male mice treated with estradiol through subcutaneous injection also had reduced cSiO2-induced lung inflammation compared to male mice that received no exogenous estradiol [169,170]. In addition, lung fibrosis was more severe in male mice compared to female mice or male mice with supplementary estradiol [169,170]. Our lab has also recently reported that male mice were susceptible to lung inflammation with both acute and sub-chronic exposure to NiNPs with or without LPS [147]. Epidemiological and animal studies suggest that sex is a critical factor in determining the susceptibility and resolution of lung inflammation. Moreover, the available literature using rodents suggests that susceptibility to acute and chronic lung disease induced by ENMs is determined in part by sex. Furthermore, epidemiology data related to ENMs are lacking due to the fact that nanotechnology is an emerging industry and human exposures are relatively new. Thus, more extensive studies should be conducted to carefully analyze the risk of ENMs to unresolved chronic lung inflammation and how the risk may differ between sexes.

## 4. Susceptible Organ Systems and Pre-Existing Disease States

### 4.1. Lung

Pre-existing lung diseases, including asthma, chronic bronchitis, COPD, or inflammation induced by microbial infection (e.g., bacterial, fungal, or viral), would likely render individuals susceptible to inhaled ENMs. As mentioned earlier, asthma affects approximately 300 million people worldwide and 26 million in the United States [21,22]. Individuals with asthma or other pre-existing lung disorders may be at a higher risk of developing more severe pulmonary disease, i.e., exacerbation, with exposure to ENMs. Our group previously reviewed the toxicology of ENMs in asthma in more detail [171]. Ovalbumin (OVA) is a commonly used allergen to induce an asthmatic phenotype in rodents, including AHR and T_H_2-mediated airway inflammation [172]. It has been shown that silica nanoparticles (SNPs) caused respiratory toxicity and exacerbated OVA-induced inflammation with higher T_H_2 cytokine levels, including IL-13 [173]. Titanium dioxide nanoparticles (TiO_2_ NPs), carbon black nanoparticles (CBNPs), MWCNTs, or zinc oxide nanoparticles (ZnONPs) have been reported to exacerbate allergic inflammatory responses in mice with increases in both T_H_1 and T_H_2 cytokines [132,174,175,176]. Collectively, these studies suggest that individuals suffering from asthma or other pre-existing respiratory disorders maybe at a higher risk for developing severe chronic lung inflammation upon exposure to ENMs. 

### 4.2. Cardiovascular

Inhalation exposure to particulate matter can lead to more severe cardiovascular conditions in individuals with pre-existing cardiovascular disorders [177]. In addition, researchers have established a relationship between occupational exposure to dust and ischemic heart diseases [178]. While there is a lack of information on cardiovascular disease and ENMs in humans, studies using rodents suggest that pulmonary exposure to ENMs could exacerbate pre-existing cardiovascular conditions in humans [179,180,181]. Additionally, several specific mediators discussed below play important roles in determining susceptibility to cardiovascular disease after ENM exposure.

Thrombospondin (TSP-1) is a protein involved in wound healing and regulating blood pressure [182,183,184,185]. TSP-1 binding to the cell surface receptor CD47 in vascular smooth muscles has been reported to inhibit endothelial nitric oxide synthase (eNOS) [182,186]. In addition, acetylcholine (ACh)-stimulated activation of eNOS leads to a decrease in blood pressure, but TSP-1 has been reported to block the Ach-stimulated decrease in blood pressure [186]. TSP-1 also has another receptor, CD36, that may also play a role in suppressing eNOS activity [183]. Thus, the impaired signaling axis of TSP-1/CD47/CD36 has been implicated in different cardiovascular diseases, including cardiac hypertrophy, impaired angiogenesis, and pulmonary hypertension [184,185,187,188,189]. Studies with *Tsp1* KO mice and *CD47* KO mice suggest that TSP-1 is critical to regulate endothelial function when exposed to ENMs [184,185]. Two studies by Mandler et al. showed that *Tsp1* KO mice exposed to MWCNTs through oropharyngeal aspiration resulted in microvascular dysregulation that could play a critical role in developing cardiovascular diseases [184,185]. This finding suggested that impairment of TSP-1 is critical to cardiovascular function and leads to greater susceptibility to severe cardiovascular disorders upon exposure to MWCNTs. 

A study using mice treated with a high dose of small, tangled MWCNTs or large, thick MWCNTs by intratracheal instillation showed a significantly higher level of serum amyloid A 3 (SAA3), haptoglobin, total cholesterol, and low-density lipoprotein in plasma [190]. The accumulation of SAA3 lipoprotein is induced by atherosclerotic plaque formation and is associated with chronic vascular inflammation [191,192]. Another study using different ENMs including MWCNTs, SWCNTs, and CB nanoparticles also showed that exposure via intratracheal instillation increased transcript level of SAA3 in the lungs in a time- and dose-dependent manner [192]. An increase in plasma proteins, along with an increased transcript level of SAA3, in mice after pulmonary exposure to MWCNTs, suggests that the risk of developing or exacerbating cardiovascular diseases could be high [190,192]. 

The low-density lipoprotein (LDL) receptor (LDLR) binds both LDL and haptoglobin [193]. A recent study used *Ldlr* KO mice to examine systemic inflammation caused by indium dioxide (In_2_O_3_) nanoparticles. After a single pharyngeal aspiration dose of In_2_O_3_ nanoparticles, *Ldlr* KO mice developed significantly severe atherosclerotic lesions compared to wild type mice [193]. The *Ldlr* KO mice also showed that the aorta had a much higher transcript level of IL-6 and MCP-1 (CCL2), suggesting ongoing inflammation in the heart [193]. *Ldlr* KO mice also showed an increase in total cholesterol and low-density lipoprotein in the plasma compared to wild type mice after treatment [193]. Therefore, this study emphasized the protective role of the LDLR in limiting cardiovascular disease after pulmonary exposure to an ENM. Moreover, these findings suggest that the pulmonary exposure to In_2_O_3_ nanoparticles can induce systemic inflammation that may exacerbate the progression of pre-existing cardiovascular diseases. 

As mentioned earlier, ApoE is a protein responsible for fat metabolism and *ApoE* KO mice represent a model for atherosclerosis in humans [116,194]. Thus, deficiency in ApoE not only results in increased susceptibility to chronic lung inflammation, but also could lead to increased susceptibility to cardiovascular diseases. A study showed that intratracheal instillation of TiO_2_ NPs significantly increased total cholesterol, nitric oxide, and eNOS in *ApoE* KO mice compared to wild type mice [195]. Another study also showed long term exposure to nickel nanoparticles (NiNPs) viawhole-body inhalation system exacerbated atherosclerosis in *ApoE* KO mice [196]. Furthermore, the study also showed that different vascular effects, including significant mitochondrial damage in the aorta and severe progression of atherosclerosis, were seen after pulmonary exposure via intratracheal instillation to carbon black nanoparticles (CBNPs) in *ApoE* KO mice [196]. Collectively, these mouse studies suggest that ApoE is likely important for suppressing cardiovascular inflammation in humans after pulmonary exposure to ENMs and that reduced ApoE would render individuals susceptible to the adverse effects of ENMs on the cardiovascular system.

### 4.3. Liver

Although few studies have investigated the systemic effects of inhalation exposure to ENMs, some work has focused on the liver to determine metabolic changes after inhalation exposure to ENMs. A recent study showed that intratracheal instillation of MWCNTs in female mice could affect liver lipid metabolism of their offspring [197]. The data showed that the weight of offspring was slightly reduced, and histopathological changes were observed in the liver tissue [197]. Intratracheal instillation of nickel oxide NPs to the lungs of rats caused pathological changes in the liver, including cellular edema and inflammatory cell infiltration, increased total nitric oxide synthase (NOS), and increased in liver-related enzymes including alanine aminotransferase (ALT), aspartate aminotransferase (AST), and alkaline phosphatase (ALP) [198]. Another study showed that sub-chronic inhalation of lead oxide nanoparticles in mice via whole-body inhalation resulted in the translocation of nanoparticles to systemic organs, including the kidney, liver, and spleen [199]. They found that lead oxide nanoparticles caused hepatic necrosis, remodeling, and degeneration of hepatocytes [199]. Furthermore, other studies have also demonstrated that inhalation exposure to silver nanoparticles, iron oxide nanoparticles, and MWCNTs results in translocation to the liver [200,201]. Repeated intratracheal instillation of TiO_2_ NPs to the lungs of rats also showed significant hepatocyte necrosis and fibrosis [202]. Furthermore, the increased presence of inflammatory cells in the liver and formation of liver fibrosis was seen in the liver through pulmonary exposure via intratracheal instillation to CBNPs in *ApoE* KO mice [202]. Therefore, these studies suggest that inhalation exposure to different ENMs has the potential to elicit systemic inflammation in the liver and could exacerbate pre-existing liver conditions such as hepatic steatosis, hepatitis, and cirrhosis. However, to our knowledge, there are no published reports on the effects of ENMs in mouse models of pre-existing liver disease.

### 4.4. Spleen

The spleen has been shown to be an important target of systemic immune responses to inhaled ENMs, specifically to MWCNTs [203,204,205]. Whole-body inhalation of MWCNTs in mice has been shown to cause systemic splenic immunosuppression [204]. Immune function was measured on spleen-derived cells and showed suppressed T cell-dependent antigen responses and reduced proliferative ability of T cells following mitogen stimulation after 14 days of MWCNTs inhalation exposure [204]. In addition, an increase in IL-10 and NAD(P)H oxidoreductase 1 mRNA level was detected only in the spleens and not in the lungs [204]. Following this study, the same group investigated the mechanism for immune suppression caused by MWCNT inhalation exposure and found that secreted transforming growth factor-beta (TGF-*β*) from the lungs had an effect on suppressing the immune function of splenocytes [203]. They demonstrated that activation of this signal in the lung from inhalation exposure to MWCNTs activated the cyclooxygenase pathways in the spleen, resulting in suppressed immune function and T cell dysfunction [203]. Another study showed that inhalation exposure to MWCNTs in rats caused immune dysfunction in the spleen [205]. After 13 weeks of exposure, rats developed systemic inflammation with increased production of inflammatory cytokines from splenic macrophages [205]. They also found a decrease in IL-2 mRNA expression in T-lymphocytes [205]. Thus, inhalation exposure to MWCNTs can affect the normal immune response generated by the spleen and could result in immunosuppression from T cell dysfunction.

### 4.5. Brain

The blood–brain barrier (BBB) consists of capillary tight junctions that selectively allow movement of specific molecules, ions, and cells between the blood and central nervous system [206]. Under healthy conditions, the BBB has capillary tight junctions that prevent particles entering the central nervous system (CNS) [206]. However, during cerebrovascular inflammation caused by inhalation of pollutants or toxicants, the tight junction of the BBB becomes destabilized with increased permeability, thereby enabling unwanted molecules or particles like ENMs to enter the CNS [207,208]. Furthermore, some studies have found that inhalation exposure to nanoparticles or ultrafine particles can reach the brain in mammals [209,210,211,212]. While such exposures that result in ENM translocation across the BBB could have deleterious effects on the CNS, some studies have shown potential benefits of using ENMs in nanomedicine to treat brain diseases [213,214,215]. Thus, the toxicity of ENMs in the brain still remains unclear. While some ENMs may not have a toxic effect on the brain, there is evidence that some ENMs elicit neuroinflammation. For example, mice exposed to MWCNTs via oropharyngeal aspiration induced BBB disruption after 4 h, which led to the recruitment of phagocytic microglia causing neuroinflammatory responses [216]. Another study also showed aerosolized exposure of MWCNTs to rats increased mitochondrial ROS formation in different parts of the brain [217]. Intranasal instillation of aluminum NPs in rats induced ERK and p38 MAPK activation in the brain, suggesting penetration through the BBB [218]. Lastly, two weeks after an intranasal instillation of AgNPs in mice, RNAseq analysis showed that 73 genes were affected in the cerebrum and 144 genes were changed in the cerebellum [219]. Therefore, these studies suggest that pulmonary exposure to different ENMs results in translocation across the BBB, causing neuroinflammation. However, the use of ENMs that are able to penetrate the BBB could also be beneficial to treat neurological disorders. More rigorous studies should be conducted in order to analyze the risk related to ENM exposure in the brain. 

## 5. Co-Exposures to ENMs and Other Agents

Co-exposures to a variety of toxic agents (e.g., metals, chemicals, microbial-derived products) could render individuals susceptible to the pro-inflammatory, pro-fibrotic, or carcinogenic effects of ENMs. For example, lipopolysaccharide (LPS) derived from Gram-negative bacteria can induce acute lung injury through neutrophilic inflammation [220,221,222]. LPS is ubiquitous in the environment and therefore, co-exposure to LPS and ENMs in occupational settings is likely [223,224,225,226]. Studies have shown that ENMs, including MWCNTs and NiNPs, can exacerbate LPS-induced lung inflammation in mice or rats [147,220]. A single co-exposure to LPS and NiNPs in mice via oropharyngeal aspiration caused significantly higher acute neutrophilic lung inflammation and greater IL-6 secretion in the BALF compared to LPS or NiNPs alone [147]. Greater acute lung inflammation induced by the co-exposure corresponded to heightened phosphorylation of STAT3 in lung tissue. Repeated co-exposure of mice to LPS and NiNPs in a sub-chronic exposure study produced greater monocytic lung inflammation and greater CCL2 in BALF compared to LPS or NiNPs alone [147]. Greater sub-chronic lung inflammation induced by LPS and NiNP co-exposure was associated with reduced STAT1 in lung tissue. MWCNT exposure has been reported to exacerbate PDGF signaling and pulmonary fibrosis in rats that were pre-exposed to LPS and had pre-existing neutrophilic lung inflammation [220]. Moreover, CBNPs also enhanced LPS-induced lung inflammation by increasing IL-1β and macrophage chemoattractant protein (MIP-1) [227]. Smaller CBNPs elicited a more profound effect by exacerbating severe LPS-induced lung inflammation [227]. Moreover, circulating fibrinogen levels were much higher in the serum of mice co-exposed to LPS and CBNPs, compared to LPS or CBNP exposure alone [227].

Proteolytic allergens from the house dust mite (HDM) *Dermatophagoides pteronyssinus* are common indoor allergens [176,228,229]. Early life exposure to HDM allergens, including Der p1 and Der p2, has been linked to the development of asthma in humans [230,231,232]. Pre-exposure of mice to HDM extract by repeated intranasal aspiration exacerbated lung inflammation and airway fibrosis caused by a single dose of MWCNTs delivered by oropharyngeal aspiration [132]. HDM-induced serum IgE levels were amplified by MWCNTs in this study [132]. In contrast, pre-exposure of mice to MWCNTs by 30 days of inhalation prior to repeated intranasal aspiration of HDM extract also exacerbated lung inflammatory lesions, but reduced HDM-induced serum IgE levels [176]. Therefore, while these two studies showed that either pre or post exposure to MWCNTs exacerbates HDM-induced chronic lung inflammation, the mechanisms involved are likely different. Overall, co-exposure to ENMs and ubiquitous allergens or LPS can exacerbate lung inflammation in rodents, suggesting that such co-exposures would increase lung disease severity in humans. 

## 6. Challenges and Alternative Approaches

Some challenges remain that must be overcome to improve risk assessment of ENMs. As the lungs are constantly exposed to a variety of inhaled toxicants and microbial agents, single ENM exposures studied in the lab using rodent models or in vitro cell culture models might not reflect a real-world exposure scenario. However, studies on co-exposures to ENMs and other inhaled toxicants or microbial agents are lacking. Therefore, more studies should be performed, using a tiered approach involving both in vitro and in vivo models to address how co-exposure to toxicants or microbial pathogens affects the immune response to inhaled ENMs. Another challenge is the knowledge gap between predicting the immunotoxicity of ENMs in rodents or humans in vivo using cell culture models in vitro. This is due, at least in part, to the complexity of the innate and acquired immune system that involves numerous cell types (e.g., macrophages, neutrophils, T cells, B cells) and subpopulations of each of these cell types. Some of these limitations could be overcome by co-culture models or ‘organ-on-a-chip’ technologies, coupled with *in silico* approaches. Furthermore, to our knowledge, no studies using mice have been performed to differentiate the immune response generated by inhalation exposure to ENMs between different age groups. As age and sex can be critical susceptibility factors in chronic lung inflammation, more studies should be done to carefully assess the toxicity of ENMs, comparing male and female mice of varying ages. This is also a challenge, as research on the immunotoxicity of ENMs is increasingly moving towards in vitro and *in silico* approaches, based on the growing number of ENMs to be evaluated and ethical concerns of using animals in research. Nevertheless, complex issues of sex and age are difficult to recapitulate using only in vitro or *in silico* approaches.

## 7. Conclusions

Herein, we have summarized different susceptibility factors, including sex, pre-existing disease state, deficiency of specific genes, susceptible organ systems, and co-exposures to other agents that might influence the pulmonary and/or systemic immune response generated by exposures to different types of ENMs. The available literature discussed in this review article illustrates that multiple factors play critical roles in determining host susceptibility to the adverse effects of ENMs on the immune system. Future research should continue to emphasize susceptibility factors and susceptible populations in risk assessments to avoid underestimating adverse outcomes in humans caused by occupational or environmental exposure to ENMs.

## Figures and Tables

**Table 1:** Summarized list of transcription factors that may be involved in the susceptibility to ENM-induced chronic lung inflammation.

Transcription Factor	Type of ENM	Dosing and Exposure Method	Duration of Exposure	Findings in KO mice	References
STAT1	Multi-Walled Carbon Nanotubes (MWCNTs) (tangled or rod-like)	4 mg/kg via oropharyngeal aspiration	Single Exposure	Increased lung fibrosis with higher TGF-β1 in bronchoalveolar lavage fluid (BALF). Increased Smad2/3 phosphorylation in lung tissue.	[82]
T-bet	Nickel Nanoparticles (NiNPs or MWCNTs	4 mg/kg via oropharyngeal aspiration	Single exposure	Enhanced mucous cell metaplasia. Increased MUC5AC and MUC5B mRNAs. Persistent eosinophils and lymphocytes in BALF. Greater interstitial lung fibrosis.	[87]
Nrf2	MWCNTs	5, 20, and 40 μg via pharyngeal aspiration	Single exposure	Higher level of inflammation and fibrosis. Increased inflammatory cell infiltrates. Increased ROS generation and oxidative damage.	[90]
	Silica NPs	10 mg/kg via intranasal instillation	Once a day for 2 weeks	Increased reactive oxygen species. Decreased total antioxidant capacity.	[91]
P53	MWCNTs (tangled or rod-like)	1 mg/kg via oropharyngeal aspiration	Once a week for 4 weeks	Increased incidence of larger granuloma formation, lymphoid aggregates, and epithelial cell hyperplasia in the lungs of heterozygous p53^(+/−)^.	[97]
BMAL1	MWCNTs	6.4 or 25.6 μg via oropharyngeal aspiration	Once a week, for 5 consecutive weeks	Increased inflammatory cytokines, oxidative stress, and procoagulant effect in serum.	[98]
	ZnONPs	6.4 or 12.8 μg via oropharyngeal aspiration	Once a week, for 5 consecutive weeks	Decreased inflammatory cytokines, decreased oxidative stress, and increased procoagulant effect.	[98]

**Table 2:** Summarized list of enzymes/proteins that may be involved in the susceptibility to ENM-induced chronic lung inflammation.

Enzymes/Proteins	Type of ENMs	Dosing and Exposure Method	Duration of Exposure	Findings in KO Mice	References
NADPH Oxidase	Single-Walled Carbon Nanotubes (SWCNTs)	40 μg/mouse via pharyngeal aspiration	Single exposure	Augmented lung inflammation by producing higher numbers of neutrophils, apoptotic cells, pro-inflammatory cytokines including TNF-α, MCP-1 (CCL2), and IL-6, and reduced anti-inflammatory cytokine, TGF-β1. Prolonged increase in neutrophils and pro-inflammatory cytokines.	[102]
COX-2	MWCNTs	4mg/kg via oropharyngeal aspiration	Single MWCNTs exposure after ovalbumin (OVA) sensitization and challenges	More susceptible to eosinophilic lung inflammation, airway mucous cell metaplasia, and airway fibrosis with ovalbumin allergen sensitization. Significantly higher Th2 cytokines including IL-13, Th1 cytokines such as CXCL10, and the Th17 cytokine IL-17A detected.	[103]
TIMP1	MWCNTs	40 µg/mouse via pharyngeal aspiration	Single exposure	Induced lung fibrosis through activation of intracellular ERK pathway	[112]
MPO	SWCNTs	40 µg/mouse via pharyngeal aspiration	Single exposure	Less efficient clearance of CNTs causing a profound inflammatory response. However, wildtype mice also showed that increased MPO was also associated with number of total cells and neutrophils.	[115]
ApoE	AuNPs C_60_ SWCNTs Carbon Black NPs (CBNPs)	0.54 µg AuNPs, 54 µg C_60_, 54 µg SWCNTs, 18 or 54 µg CBNPs via instillation or inhalation	Single exposure	Increased the DNA damage of inflammatory cells, neutrophil percentage, and higher protein level in BALF.	[119]
	MWCNTs	4 or 40 μg/mouse via intratracheal instillation	Once a week for 4 weeks	Increased pulmonary inflammation and oxidative stress/damage to DNA in lung tissue.	[120]
	SWCNTs Or Double-Walled CNTs	10 or 40 μg/mouse via pharyngeal aspiration	Once every other week for 10 weeks	Dysregulation of endothelial progenitor cell (EPC) function contributing to developing atherosclerosis, buildup of cholesterol plaques in the walls of arteries.	[121]
	MWCNTs	6.4 or 25.6 μg/mouse via intratracheal instillation	Once a week for 5 weeks	More susceptible to oxidative damage to DNA in lung tissue. Accelerated progression of atherosclerosis.	[122]

**Table 3:** Summarized list of receptors and cytokines/chemokines that may be involved in the susceptibility to ENM-induced chronic lung inflammation.

Cytokines/Chemokines	Type of ENMs	Dosing and Exposure Method	Duration of Exposure	Findings in KO Mice	References
IL1/Inflammasome	MWCNTs	50 µg low nickel or high nickel containing MWCNT via oropharyngeal aspiration	Single exposure	Reduced acute inflammation and airway resistance but increased IL-6 protein production within 1 day.	[133]
	
				Induced significantly higher number of pulmonary granulomas formation and significant inflammation post 28 days.	
		162 μg/mouse Mitsui-7 MWCNTs via intratracheal instillation	Single exposure	Acute inflammation at day 1 was suppressed. Fibrotic lesions still developed in KO mice 28 days post exposure.	[134]
		10 µg rod-like MWCNTs for 4hr via pharyngeal aspiration 10 and 40 µg rod-like MWCNTs for 28 days via pharyngeal aspiration	Single exposure	Reduced neutrophils in BALF and neutrophil chemoattractant CXCL5 mRNA levels 4hr after the exposure. Neutrophils were still reduced in BALF and TNF-α mRNA level was suppressed after 28 days. No changes were observed in Th2 related signals including IL-13 and TGF-β1 mRNA levels.	[135]
OPN	MWCNTs	40 μg via pharyngeal aspiration	Single exposure	Reduced fibrotic formation and myofibroblast accumulation in the lungs.	[136]
AhR	ZnONPs	5, 20, and 80 μg/mice via oropharyngeal aspiration	Single exposure	Reduced pulmonary inflammation, cytokine secretion, CYP1A1, and KYN production. Reduced cell number, total protein, and LDH activity in BALF.	[125]
CCR5	SWCNTs	100 μg/kg via intratracheal instillation	Single exposure	Dominated by B cells and CD8+ T cells instead of T cells and CD4+ T cells in the lungs. Increased IL-6, IL-13, and IL-17 in BALF. More frequent histopathological lesions were detected.	[128]
